# Research on cross-regional emergency materials intelligent dispatching model in major natural disasters

**DOI:** 10.1371/journal.pone.0305349

**Published:** 2024-07-26

**Authors:** Lin Zhang, Jinyu Wang, Xin Wang, Wei Wang, Xiangliang Tian

**Affiliations:** 1 School of Economics and Management, Beijing Information Science and Technology University, Beijing, China; 2 Fire Fighting Theory Laboratory, Shanghai Fire Science and Technology Research Institute of MEM, Shanghai, China; 3 Key Laboratory of Non-coal Mine Safety Risk Monitoring and Early Warning National Mine Safety Administration, China Academy of Safety Science and Technology, Beijing, China; University of Botswana, BOTSWANA

## Abstract

The increasingly frequent occurrence of major natural disasters can pose a serious threat to national stability and the safety of people’s lives, and cause serious economic losses. How to quickly and accurately dispatch emergency materials to all disaster areas across regions in post-disaster has attracted wide attention from the government and academia. In response to the characteristic of high uncertainty in emergency rescue for major natural disasters, and considering differentiated disaster severity levels in different disaster areas, the entropy weight method is used to determine the urgency coefficient of emergency material demand for disaster areas. This study aims to minimize the emergency materials dispatching time and cost, also maximize the dispatching fairness for disaster areas. The triangular fuzzy number method is used to represent the uncertain variables mentioned above, so that a cross-regional emergency materials intelligent dispatching model in major natural disasters (CREMIDM-MND) is constructed. The extremely heavy rainstorm disaster in Henan Province of China in 2021 is selected as a typical case. Based on objective disaster data obtained from official websites, this study applies the constructed model to real disaster case and calculates the results by MATLAB. The ant colony algorithm is further used to optimize the transportation route based on the calculation results of the emergency material dispatching for disaster areas, and finally forms the intelligent emergency materials dispatching scheme that meets the multiple objectives. The research results indicate that compared to the actual situation, CREMIDM-MND can help decision-maker to develop a cross-regional emergency materials intelligent dispatching scheme in time, thereby effectively improving the government’s emergency rescue performance in major natural disasters. Moreover, some managerial insights related to cross-regional emergency materials dispatching practice problem in major natural disasters are presented.

## 1. Introduction

Major natural disasters occur frequently around the world, not only posing a threat to national stability and people’s safety, but also causing serious economic losses [1]. China is one of the countries with the most severe natural disasters in the world, characterized by a wide variety of disasters, wide distribution area, high frequency of occurrence, and heavy losses. Especially in recent years, the suddenness and destructiveness of natural disasters have become increasingly obvious [2]. How to effectively coordinate the emergency material demand of each disaster area and the emergency material reserves of each rescue area within the shortest time in major natural disasters. So that to make more effective, economic, and fair emergency material dispatching schemes, timely complete the transportation of emergency materials, and ultimately improve the emergency response performance of government departments. This important issue needs for urgent discussion.

At present, scholars have conducted extensive research on a series of problems in emergency materials dispatching, such as multi-objective optimization, fuzzy optimization, path optimization, etc. For example, Liu et al. [3] argued that balancing multiple objectives (such as risk, time, and cost) in emergency rescue is beneficial for improving the effectiveness of emergency schemes. Therefore, a hybrid method combining dynamic Bayesian network with graphical evaluation and review technique for evaluating and optimizing emergency schemes is proposed. Wan et al. [4] proposed that large-scale emergencies usually cause extensive damage to equipment and facilities, and even communication interruptions, which ineluctably results in the difficulty of obtaining relevant disaster information (e.g., the casualties, the demands of disaster areas, road destructions, etc.). Thus, it’s reasonable to concern more uncertain or fuzzy information for making decisions of emergency material dispatching. For this reason, a bi-objective trapezoidal fuzzy emergency distribution center location model which reflecting the urgency and uncertainty of major natural disasters is established. Since major natural disasters have considerable effects on transportation networks, transportation route optimization for emergency materials dispatching plays an important role in the process of emergency rescue. Nikoo et al. [5] presented a three-objective model to identify the optimal routes for emergency vehicles considering the length, the travel time and the number of routes.

It is worth noting that due to the fact that the coverage of most major natural disasters usually exceeds the jurisdiction of a single region, the emergency materials reserve within the region often results in the difficulty of meeting the demand in disaster area, which requires cross-regional coordination in response to disasters [6]. Although government departments have put this proposal into practice, this study finds that there are still challenges in cross-regional emergency materials dispatching in major natural disasters. 1) Time is life, particularly faced with a severe disaster. Successful emergency rescue should meet the requirements of effectiveness in the shortest time [7]. Compared to normal scenario, emergency material dispatching in major natural disasters often exhibits some unique challenges such as degraded transportation infrastructures, unoptimistic weather conditions, insufficient material reserves in disaster areas, and difficulty in obtaining emergency rescue information. All these challenges will cause delays in emergency materials transporting [8]. Therefore, it’s more difficult to control the total dispatching time within the shortest possible range. 2) Scholars point out that the cost of emergency material dispatching should not be ignored while considering the consuming of dispatching time in major natural disasters, since the economic expenditure is tighter than usual [9]. However, cross-regional emergency rescue for major natural disasters often faces geographical dispersion. The increase in emergency materials dispatching distance and the use of a large number of fleets will lead to an enhance in dispatching cost [10]. Thus, it’s difficult to make a trade-off between time efficiency and economic cost. 3) As is known for all, there will be differences of disaster severity in different disaster areas follow a major natural disaster, which leads to different demand urgency of emergency materials in each disaster area. In order to achieve the fairness of emergency materials dispatching, reasonable coordinating and dispatching emergency materials according to the demand urgency of each disaster area, which plays an important role in improving the effectiveness of emergency rescue [11]. However, the “cost” and “response speed (i.e., urgency)” of post-disaster emergency rescue activities often arise the confliction in decision-making [12]. Therefore, how to balance the “cost”, “time”, and “urgency” of cross-regional emergency materials dispatching in major natural disasters is a major challenge faced by post-disaster emergency rescue. Based on the above, this study attempts to answer the questions: “What is the appropriate method for cross-regional emergency material dispatching in major natural disasters? How to balance dispatching time, cost, and fairness when facing multiple disaster area?”

Although previous studies have their merits in improving the efficiency in terms of emergency material dispatching, there are still several limitations on cross-regional emergency material dispatching in major natural disasters. On the one hand, some studies aim to minimize the total transportation time and construct optimization models for emergency material dispatching, but less consideration is given to the impact of differentiated disaster severity levels on emergency material dispatching [13–15]. Some studies consider the demand urgency of disaster areas and established emergency logistics models, but they have not taken into account the fairness of emergency material dispatching while considering differentiated disaster severity levels [16, 17]. On the other hand, some studies assume that the environment of emergency material dispatching is deterministic, and the parameters in the model are taken as certain numbers [18, 19]. In fact, a high degree of uncertainty usually exists in major natural disasters, which cannot help in making effective emergency decisions. To fill the above research gaps, this study comprehensively takes account into the challenges of emergency rescue in major natural disaster and explores the multi-objective optimization problem of cross-regional emergency materials dispatching. The primary contributions of this study are as follows:

- Before making the emergency materials dispatching scheme, the principle of proximity is not used to allocate the emergency materials. Instead, the entropy weight method is used to determine the urgency coefficient of emergency material demand of multiple disaster area under major natural disasters.
- A multi-objective optimization model for cross-regional emergency materials intelligent dispatching is proposed, which aims to minimize dispatching time, minimize dispatching cost, and maximize dispatching fairness. Triangular fuzzy numbers and ant colony algorithm are used to make emergency materials dispatching decisions, with the aim of improving the government’s emergency rescue performance.
- A case study is conducted to examine the feasibility of the adopted method. The comparison between the research results and the actual demand demonstrates the effectiveness of the model constructed in this study, which significantly reducing dispatching time and dispatching cost, and enhance the fairness of emergency rescue to disaster areas.

The remainder of this paper is organized as follows. Section 2 analyzes the literature on the aspects of emergency materials dispatching and transportation route optimization in major natural disasters, and identifies the research gap from existing studies. Section 3 constructs CREMIDM-MND with the objectives of minimizing dispatching time and cost, and maximizing dispatching fairness to multiple disaster areas. In Section 4, this study applies the constructed model to a typical case of major natural disaster which happened in China. By comparing and discussing the computational results through the model with real data, the feasibility of the adopted method and the effectiveness of the constructed model is verified. Section 5 concludes the paper with some remarks as well as limitations and future research directions.

## 2. Literature review

Emergency rescue is a very important process of emergency management in major natural disasters. Timely and on-demand dispatch of emergency materials to disaster areas is a key task of emergency rescue. Therefore, the academic community has conducted extensive research on this issue and obtained a large number of good research results. In recent years, research on cross- regional emergency materials dispatching in major natural disasters has mainly focus on two aspects: 1) research on emergency materials dispatching, 2) research on transportation route optimization. This section provides a review on these two issues.

### 2.1 Emergency materials dispatching

As an important component of the response phase of post-disaster emergency rescue activities, emergency materials dispatching has become an active research field in recent years [20]. In the face of major natural disasters, efficient emergency materials dispatching is a necessary and essential condition for saving lives and reducing losses [21]. However, as a type of emergencies, major natural disasters, due to the characteristics of the suddenness, urgency of time, and the combined effects of various subjective and objective factors, may result in decision-makers being uncertain, and insufficient access to disaster information. Failure to make timely and correct emergency responses can have a negative impact on emergency materials dispatching, and even let the disasters develop into a more significant catastrophic event [22]. To address this issue, scholars have conducted in-depth research on three aspects, including emergency materials dispatching model construction, the uncertainty factors of emergency rescue, and the urgency of emergency materials demand in disaster areas. For example, Ahmadi et al. [23] presented a multi-depot location-routing model considering network failure, multiple uses of vehicles, and standard relief time, which significantly reduce the dispatching time of emergency materials. Ferrer et al. [24] built a multi-criteria optimization compromise programming model for humanitarian last mile emergency materials distribution in the aftermath of disasters. Sabouhi et al. [25] constructed an integrated routing and scheduling model for evacuation and commodity distribution in large-scale disaster. Dalal and Uster [26] developed an optimization model for determining centralized supply locations, and supply quantities under different transportation modes to respond timely for foreseen disasters. Khanchehzarrin et al. [27] proposed a bi-level multi-objective location-routing optimization model for disaster relief operations considering the dispatching cost, time, efficiency and the supply risk.

The complex nature of real-world problems will generate a large number of uncertainty issues [28]. Thus, introducing an effective method to make reasonable estimation of the uncertain information is one of the key factors in improving the effectiveness for making emergency decision [29]. Triangular fuzzy number is often used to represent uncertain data in the process of emergency materials dispatching due to the characteristic of easy to express and the function of handling random, fuzzy, insufficient, or imprecise data [30]. Fazayeli et al. [31] argued that due to vagueness of real-world data, triangular fuzzy numbers can better express requirements. Therefore, emergency materials demand is represented by triangular fuzzy numbers. Wan et al. [4] proposed a multi-period dynamic emergency material distribution model, which uncertain demand and transportation time are described by triangular fuzzy numbers to better fit with the real situation.

In addition, in response to the reality of differences in the urgency of emergency materials demand in different disaster areas, multiple criteria decision analysis methods such as entropy weight method, analytic hierarchy process (AHP) method, and the method of technique for order performance by similarity to ideal solution (TOPSIS) are commonly used to solve this problem. For example, Guan et al. [32] forecast the emergency materials demand in post-disaster, and the entropy weight-TOPSIS method is used to measure the urgency of affected areas, thereby constructing a multi-objective collaborative emergency material dispatching model. Geng et al. [33] comprehensively used the methods of fuzzy AHP, fuzzy TOPSIS, and multi-objective weighted optimization to optimize the location and quantity of emergency materials. Thereinto, the fuzzy TOPSIS method was used to evaluate the urgency of emergency materials demand in disaster areas.

In view of this, it’s of great significance to use scientific methods to characterize the uncertainty factors in the process of emergency materials dispatching and accurately evaluate the urgency of emergency materials demand in disaster areas, which is crucial for constructing CREMIDM-MND.

### 2.2 Transportation route optimization

Transportation route optimization problems have the characteristic of dispatching limited vehicles to accomplish transportation tasks in rigidly limited times and harsh environment conditions [34]. In emergency rescue, transportation route optimization is related to whether the emergency materials dispatching efficiency can be enhanced, so as to reduce the life and property losses caused by disasters [35]. Therefore, as an important module for emergency materials dispatching, research on transportation route optimization in disasters has received continuous attention from scholars for many years. For example, Chai et al. [36] proposed a route optimization model based on the relationship between the total routing length and queuing delays to reduce the decision time and improve the efficiency of rescue, which makes the transportation route more practical. Yi et al. [37] constructed an optimization model of transportation route problems with emergency materials in sudden disasters to reach the best solution in the least amount of time. Xu et al. [38] proposed an efficient transportation route planning scheme based on the mobile cloud computing paradigm, which can be able to support large-scale emergency-management scenarios. Liu et al. [39] construct a multi-objective model of emergency materials transportation routes optimization for large cities in case of an epidemic outbreak, which aimed to achieve the objectives of highest vehicle utilization rate and lowest transportation cost. These studies provide theoretical support and methodological inspiration for the transportation route optimization of emergency materials dispatching. On this basis, in-depth exploration is conducted on how to use advanced methods to solve the transportation route problems, in order to further improve the of transportation efficiency in the process of emergency materials dispatching.

The existing studies show that high-performance algorithms can significantly enhance the likelihood of obtaining the optimal transportation route scheme for solving transportation route problems in emergency materials dispatching [40, 41]. Ant colony algorithm, as a heuristic global optimization algorithm in evolutionary algorithms, has the characteristics of distribution calculation, positive feedback of information, and heuristic search. Thus, it’s often used to solve transportation route optimization problems [42, 43]. Wang et al. [44] constructed an optimal transportation routing model intending to minimize the combined travel time of all emergency vehicles during post-disaster scenarios, and a hybrid ant colony optimization algorithm was used to solve the emergency transportation problems. Wan et al. [45] established a multi-objective multi-constraint emergency material dispatching model based on the principle of ordered arrival, which a hybrid ant colony optimization was proposed to solve transportation route optimization problems. Ferrer et al. [46] developed an elaborated methodology based on ant colony optimization metaheuristic for solving multi-criteria last mile transportation routing optimization problems to provide good quality solutions in a short time. Liu et al. [47] argued that the speed, cost and benefit of vehicle transportation were affected by the route to a certain extent. For this reason, they built an intelligent model based on improved ant colony algorithm to optimize the emergency materials transportation route.

Based on the above, it can be seen that in the transportation route optimization problems, the ant colony algorithm can help to obtain the optimal transportation route scheme, so as to effectively improve the transportation efficiency, shorten the transportation time and reduce the transportation cost in within the process of emergency materials dispatching.

### 2.3 Research gap

Emergency materials dispatching and transportation route optimization problems are interdisciplinary research fields involving operations research, logistics, and computer science. The final objectives are to quickly and effectively organize emergency materials dispatching activities, optimize transportation route, and minimize losses caused by major natural disasters [48, 49]. Although scholars have conducted extensive research on these issues, there are still some research gaps: a) when major natural disasters occur, they are often accompanied by the scenario of unpredictable risks and information uncertainty. Further in-depth research is needed on how to make reasonable emergency materials dispatching decision in situations where information is incomplete and demand is uncertain [50]. b) Previous studies on emergency materials dispatching and transportation route optimization often focused on decision-making at a single scale (such as within cities or regions) or with a single objective (time efficiency or economical efficiency) [17]. However, disaster emergency response usually requires decision-making integration of multiple scales (such as adjacent cities or across regions) and multiple objectives (such as time, cost, and fairness). Therefore, research on this area needs to be further explored.

Although research on emergency materials dispatching and transportation route optimization has shown a rapid growth trend in recent years, it’s still in the exploratory phase. Especially in the case of major natural disasters, the issue of cross-regional emergency materials dispatching has not yet achieved a good organic coordination and integration of materials dispatching and transportation route optimization. Considering uncertainty characteristic of emergency rescue in major natural disasters, this study seeks the optimal solution among multiple conflicting objectives include of reducing dispatching time and dispatching cost, and ensure the dispatching fairness for all disaster areas. CREMIDM-MND based on the combination of entropy weight method, triangular fuzzy number and ant colony algorithm is proposed, which aims to provide support for improving the government emergency rescue performance.

## 3. Model and methods

**Fig 1:**
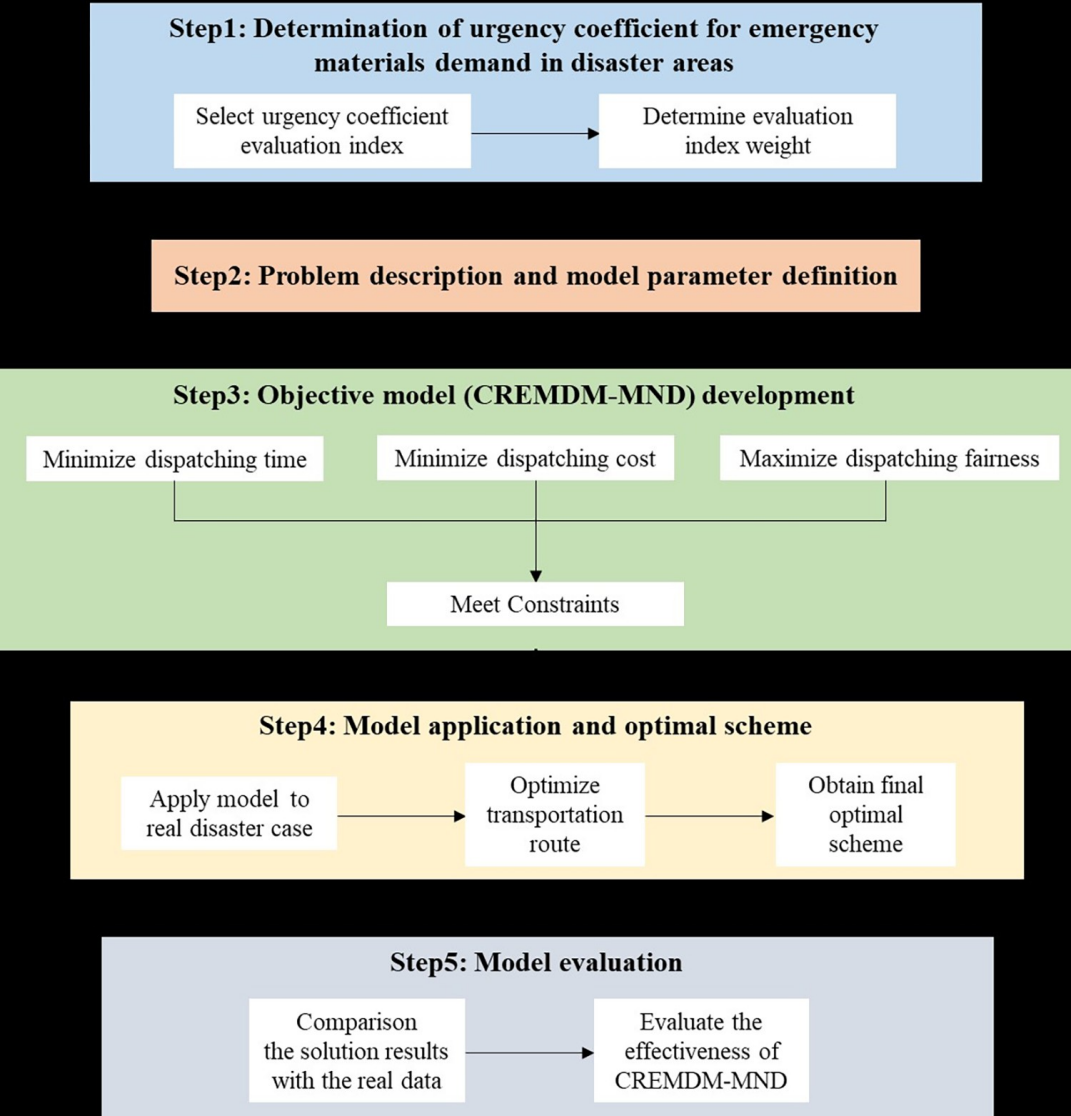
Model and methods framework.

Fig 1 depicts the framework for model and methods. Step1 determines the urgency coefficient of emergency materials demand in disaster areas. Step2 descripts the problem and defines the parameter symbols in the model. Step3 constructs CREMIDM-MND with the multiple objectives of minimum dispatching time, minimum dispatching cost and ensure maximum dispatching fairness. Step 4 applies CREMIDM-MND to real disaster case and seeks the optimal transportation route based on the solution results to obtain the final dispatching scheme. Step5 evaluates the performance of the model by comparing the solution results with the real data.

### 3.1 Determination of urgency coefficient for emergency materials demand in disaster areas

Due to the suddenness and uncertainty of major natural disasters, the disaster degree of each affected area will be different. Therefore, the urgency of emergency materials demand varies among different disaster areas. If emergency materials are only dispatched based on the principle of proximity, it may lead to delay of emergency rescue in severely disaster areas, resulting in more serious consequences. For this reason, considering the differences in the urgency of emergency materials demand among disaster areas to reasonably arrange emergency rescue tasks is conducive to providing support for the formulation of emergency materials dispatching scheme, so as to improve the effectiveness of emergency rescue.

#### 3.1.1 Selection of urgency coefficient evaluation index for emergency materials demand

On the basis of the previous studies and considering the availability of data, 5 accuracy indexes are selected from the 28 basic indexes of disaster statistics in China’s national standard “GB-T/24438.1–2009” to evaluate the urgency coefficient of emergency materials demand for disaster areas in this study [51]. The 5 indexes are as follows:

1. Disaster-affected population. It refers to the number of people who suffered losses due to natural disasters. The more the affected population, the more serious the disaster, and the higher the urgency of the emergency materials demand.
2. Dead and missing population. It refers to the number of people who dead or missed for as a direct result of natural disasters. The more dead and missing, the more serious the disaster, and the higher the urgency of the emergency materials demand.
3. Emergency transfer and resettle population. It refers to the number of people who are transferred and resettled from disaster areas to safe areas due to the threat of natural disasters. The more transfer and resettle population, the more serious the disaster, and the higher the urgency of emergency materials demand.
4. Number of damaged houses. The number of houses that need to be repaired due to natural disasters causing damage to load-bearing components, or obvious cracks in non-load-bearing components, or damage to ancillary structures. The more damaged houses, the more serious the disaster, and the higher the urgency of the emergency materials demand.
5. Direct economic loss. It refers to the total amount of material property value reduction or loss directly caused by natural disasters. The higher the amount of direct economic loss, the more serious the disaster, and the higher the urgency of the emergency materials demand.

#### 3.1.2 Determination of evaluation index weight based on entropy weight method

The entropy weight method is originally developed from the information entropy theory which proposed by American scholar Shannon. Its basic idea is that when the probability of each event occurring is the same, the uncertainty of information is maximum, and this degree of uncertainty is called entropy. Conversely, when the probability of some events is higher than others, the uncertainty of the information will decrease, and the entropy will decrease accordingly. By calculating the entropy value of each index and converting it into weight coefficients, quantitative comparison and comprehensive evaluation between indexes can be achieved, which is a commonly used multi-index comprehensive evaluation method [52].

The specific calculation steps of this method are as follows:

1. Establishing the indexes matrix
  Set the set of m disaster areas as *D* = {*D*_1_, *D*_2_,⋯,*D*_*m*_}, each disaster area in the set has an evaluation index. Let *d*_*if*_ be the *f-th (f = 1*,*2*,⋯,*a)* impact factor index data of the *i-th* disaster area *D*_*i*_, then the index data matrix is
$$D={\left(d_{if}\right)}_{m\times a}=\left[\begin{array}{ccc} d_{11} & \cdots & d_{1a}\\ \cdots & & \cdots \\ d_{m1} & \cdots & d_{ma} \end{array}\right]$$ (1)
2. Standardization of the indexes
  Since the dimensions of all indexes are different, the data cannot be directly compared, so the data needs to be standardized, and the standardized value is
$$d_{ij}^{*}=\frac{d_{if}-min\{d_{1f},d_{2f}{,\cdots ,d}_{nf}\}}{max\{d_{1f},d_{2f}{,\cdots ,d}_{nf}\}-min\{d_{1f},d_{2f}{,\cdots ,d}_{nf}\}}$$ (2)
3. Normalization of the indexes matrix
  In order to eliminate the impact of the index measurement unit, the evaluation index should be normalized.
$$p_{if}=\frac{d_{if}^{*}}{\sum_{i=1}^{m}d_{if}^{*}},i=1,2,\cdots ,m;f=1,2,\cdots ,a$$ (3)
$$P_{if}={\left(p_{if}\right)}_{m\times a}\left[\begin{array}{ccc} p_{11} & \cdots & p_{1a}\\ \cdots & & \cdots \\ p_{m1} & \cdots & p_{ma} \end{array}\right]$$ (4)
4. Calculation of index’s information entropy
$$E_{f}=-k\sum_{i=1}^{a}\left(p_{if}\ln\left(p_{if}\right)\right)$$ (5)
  In the formula, the constant $k=\frac{1}{\ln\;m};\;0\leq E_{f}\leq 1$, that is, the maximum value of *E*_*f*_ is 1.
5. Calculation of index’s information entropy redundancy
$$e_{f}=1-E_{f}$$ (6)
  When *e*_*f*_ = 0, the f-th evaluation index can be eliminated, and its weight is equal to 0.
6. Calculation of the index’s entropy weight
$$W_{f}=\frac{e_{f}}{\sum_{f=1}^{a}e_{f}},f=1,2,\cdots ,a$$ (7)
7. Determination of urgency coefficient
  The general weighted summation method is used to determine the urgency score of emergency materials demand in each disaster area. Based on the urgency score of each disaster area, the disaster area with the lowest score is used as the benchmark, and the scores of all disaster areas are compared to obtain the relative urgency coefficient of each disaster area, as shown follows.
$$Q_{i}=\sum_{f=1}^{n}W_{f}\times p_{if},i=1,2,\cdots ,m$$ (8)
$$\lambda_{i}=\frac{Q_{i}}{Q_{min}},i=1,2,\cdots ,m$$ (9)
  Among them, *Q*_*i*_ is the urgency score of the demand for the disaster area *i*; *λ*_*i*_ is the urgency coefficient of the disaster area *i*; *Q*_*min*_ is the minimum urgency coefficient score of emergency materials demand for the disaster areas.

### 3.2 Problem description

Assumption *D*_*i*_(*i* = 1,2,⋯,*m*) is the disaster area, and *S*_*j*_(*j* = 1,2,⋯,*n*) is the emergency materials rescue area. Among them, there are *R*(*R*>1) types of emergency materials that need to be dispatched to disaster area *D*_*i*_, and *k*(1≪*k*≪*R*) represents the *k-th* type of materials.

It is known that the emergency materials storage capacity of rescue area *S*_*j*_ is *s*_*j*_. The actual supply quantity of *k-th* emergency material *R*_*k*_ from the rescue area *S*_*j*_ to the disaster area *D*_*i*_ is $x_{ij}^{k}$. The demand quantity for the *k-th* emergency material in the disaster area *D*_*i*_ is $d_{i}^{k}\;(d_{i}^{k}>0)$. The storage capacity of *k-th* emergency material in the rescue area *S*_*j*_ is $s_{j}^{k}$. The dispatching unit cost is *c*_*ij*_, and the dispatching time is *t*_*ij*_. The decision variable *y*_*ij*_∈(0, 1) is used to represent the emergency materials dispatching situation from rescue area *S*_*j*_ to disaster area *D*_*i*_. When dispatching emergency materials from rescue area *S*_*j*_ to disaster area *D*_*i*_, set *y*_*ij*_ = 1, otherwise *y*_*ij*_ = 0.

There is usually a shortage of emergency materials in the early stages of emergency rescue. To achieve the objectives of minimizing dispatching time and dispatching cost, and maximizing the dispatching fairness for all disaster areas, it’s necessary to develop the optimal emergency materials dispatching scheme. Therefore, it should be calculated the types and quantities of emergency materials supplied by each rescue area to the corresponding disaster areas as soon as possible.

### 3.3 Model

In early stage of emergency rescue in major natural disasters, due to the complex geographical environment as well as the fact that disaster information often has a series of characteristics such as uncertainty, complexity, and fuzz, the triangular fuzzy number method can help decision-makers to make the effective decisions. Therefore, this study uses triangular fuzzy number method to represent the uncertainty of the emergency materials demand in different disaster areas and the uncertainty of the dispatching time and dispatching cost. The triangular fuzzy number of emergency materials demand in the disaster area *D*_*i*_ is represented as $\tilde{d_{i}^{k}},\;\tilde{d_{i}^{k}}=(d_{i1}^{k},d_{i2}^{k},d_{i3}^{k})$; the triangular fuzzy number of dispatching time from rescue area *S*_*j*_ to disaster area *D*_*i*_ is represented as $\tilde{t_{ij}},\;\tilde{t_{ij}}=(t_{ij1},t_{ij2},t_{ij3})$; the triangular fuzzy number of dispatching cost from rescue area *S*_*j*_ to disaster area *D*_*i*_ is represented as $\tilde{c_{ij}},\;\tilde{c_{ij}}=(c_{ij1},c_{ij2},c_{ij3})$. According to the imported triangular fuzzy number of emergency materials demand, dispatching time and dispatching cost, the optimal emergency material dispatching scheme is developed to better achieve the objectives of the minimum dispatching time and dispatching cost, and the maximum dispatching fairness.

#### 3.3.1 Timeliness objective function (Minimum dispatching time)

After a major natural disaster, the primary goal to be met is to minimize the emergency materials dispatching time, see Formula (10):

$$minf_{1}(x)=\sum_{i=1}^{m}\sum_{j=1}^{n}\tilde{t_{ij}}y_{ij}$$ (10)

Among them, $\tilde{t_{ij}}$ represents the triangular fuzzy number of dispatching time from rescue area *S*_*j*_ to disaster area *D*_*i*_, in minutes, *y*_*ij*_ indicates whether the rescue area *S*_*j*_ provides emergency materials to the disaster area *D*_*i*_, if so, set *y*_*ij*_ = 1, otherwise *y*_*ij*_ = 0.

#### 3.3.2 Economic objective function (Minimum dispatching cost)

The primary goal of emergency material dispatching is to dispatch the emergency materials required by each disaster area from the rescue area in the shortest possible time. Under this premise, further realize to minimize the dispatching cost, see Formula (11):

$$minf_{2}(x)=\sum_{k=1}^{l}\sum_{i=1}^{m}\sum_{j=1}^{n}x_{ij}^{k}y_{ij}\tilde{c_{ij}}$$ (11)

Among them, $x_{ij}^{k}$ represents the actual supply quantity of the *k-th* emergency material *R*_*k*_ from the rescue area *S*_*j*_ to the disaster area *D*_*i*_, *y*_*ij*_ indicates whether the rescue area *S*_*j*_ provides emergency materials to the disaster area *D*_*i*_, if so, set *y*_*ij*_ = 1, otherwise *y*_*ij*_ = 0. $\tilde{c_{ij}}$ represents the triangular fuzzy number of the dispatching cost of rescue area *S*_*j*_ to disaster area *D*_*i*_.

#### 3.3.3 Fairness objective function (Maximum dispatching fairness)

In order to avoid blindly pursuing the minimum dispatching time and dispatching cost, resulting in the situation of uneven emergency materials dispatching in each disaster area, it’s necessary to ensure that the unsatisfied rate of emergency materials dispatching in each disaster area to be the lowest. That is, the dispatching fairness of all disaster areas is required to be the best [53], as shown in Formula (12):

$$minf_{3}(x)=max\left(\lambda_{i}(1-\sum_{j=1}^{n}\sum_{k=1}^{l}\frac{x_{ij}^{k}}{\sum_{k=1}^{l}\tilde{d_{i}^{k}}})\right)$$ (12)

In the formula, *f*_3_(*x*) is the lowest unsatisfied rate of emergency material demand in each disaster area. $\tilde{d_{i}^{k}}$ is the triangular fuzzy number of the disaster area *D*_*i*_’s demand for the *k-th* emergency material. *λ*_*i*_ is the urgency coefficient of emergency materials demand in the disaster area.

#### 3.3.4 Constraints

The model constructed in this study includes three objective functions: the minimum dispatching time (10), the minimum dispatching cost (11) and the maximum dispatching fairness (12). The constraints are:

s.t.


$$\sum_{j=1}^{n}x_{ij}^{k}y_{ij}\leq \tilde{d_{i}^{k}};(i=1,2,\cdots ,m)$$ (13)

$$\sum_{i=1}^{m}x_{ij}^{k}y_{ij}\leq s_{j}^{k};(j=1,2,\cdots ,n)$$ (14)

$$\frac{\sum_{j=1}^{n}x_{ij}^{k}y_{ij}}{\tilde{d_{i}^{k}}}\geq 80\%;(i=1,2,\cdots ,m)$$ (15)

$$x_{ij}^{k}\geq 0$$ (16)

$$y_{ij}\in \{0,1\};\;i=1,2,\cdots ,m,j=1,2,\cdots ,n$$ (17)

$$\left\{ \begin{array}{c} x_{ij}^{k}>0,y_{ij}=1\\ x_{ij}^{k}=0,y_{ij}=0 \end{array}\right.$$ (18)

$$\sum_{i=1}^{m}y_{ij}\geq 1$$ (19)

Eq (13) indicates that the actual demand for emergency materials obtained by disaster area *D*_*i*_ does not exceed its own fuzzy demand.

Eq (14) indicates that the total quantity of all types of emergency materials provided by rescue area *S*_*j*_ is not greater than its own reserves.

Eq (15) indicates that the minimum satisfaction rate of actual emergency materials obtained by disaster area *D*_*i*_ is not less than 80%.

Eq (16) indicates that the non-negative constraint on the dispatching quantity of emergency materials.

Eq (17) indicates that the decision variable *y*_*ij*_ only takes 0 or 1.

Eq (18) indicates whether emergency materials are dispatched from *S*_*j*_ to *D*_*i*_. If emergency materials are dispatched from *S*_*j*_ to *D*_*i*_, *y*_*ij*_ = 1, otherwise 0.

Eq (19) indicates that at least one rescue area has provided emergency materials for each disaster area.

## 4. Case study

### 4.1 Case description

From July 17 to 23, 2021, China’s Henan Province suffered from a rare extremely heavy rainstorm disaster in history, causing heavy casualties and property losses, and many regions in Henan Province were severely affected. Therefore, this disaster is selected as a typical case of major natural disasters in this study. The 6 most severely affected cities Zhengzhou, Gongyi, Xinmi, Xinzheng, Xingyang, and Dengfeng in Henan Province are selected as the disaster areas in this study, which are represented by *D*_1_, *D*_2_, *D*_3_, *D*_4_, *D*_5_ and *D*_6_ respectively.

There are usually many uncertain factors in the case of sudden disasters. In order to be more realistic, the following assumptions are proposed in this study:

1. The emergency materials selected in this study are mainly general materials for disaster relief, such as drinking water, food and masks (during COVID-19).
2. The emergency materials required by each disaster area are multiple types.
3. The fuzzy demand for emergency materials in each disaster area is roughly calculated based on the disaster degree and disaster-affected population data.
4. The dispatching time and dispatching cost of emergency materials is calculated based on the real-time search data of Amap App.
5. The dispatching cost of emergency materials is set as the transportation cost of emergency materials, that is, the sum of the load charge of the transportation vehicle and the toll of the transportation vehicle.
6. Each transportation vehicle only undertakes one task of transporting emergency materials, and returns to the original rescue area after completing the task, regardless of the return time.
7. The carrying capacity of each transportation vehicle is set at 5 tons, and the average speed of the vehicle is equal.
8. The preparation time of rescued emergency materials in each rescue area and the unloading time of receiving emergency materials in each disaster area are ignored.
9. The data of emergency materials demand in each disaster area is obtained from the official website of the relevant department, and has been processed to a certain extent without losing authenticity [54].

Since the bottled water and instant noodles are usually the immediate necessities for daily life after disaster, while masks are the necessary material for prevention and control the epidemic during COVID-19, these three kinds of emergency materials all belong to the general materials for disaster relief. Moreover, bottled water, masks, and instant noodles are the three types of emergency materials with the highest demand in this disaster. Therefore, these three kinds of emergency materials are selected as examples in this study, which are represented by *R*_1_, *R*_2_ and *R*_3_ respectively. Based on the above assumptions and according to the population size of each disaster area, this study sets the triangular fuzzy number of emergency materials demand in each disaster area, as shown in Table 1.

**Table 1:** Triangular fuzzy number of emergency materials demand in each disaster area.

$\tilde{d_{i}^{k}}$	*R*_1_	*R*_2_	*R*_3_
*D*_1_	(78 600, 110 000, 150 000)	(115 000, 230 000, 345 000)	(22 000, 42 000, 84 000)
*D*_2_	(1 000, 1 400, 2 000)	(8 000, 16 000, 24 000)	(280, 550, 1 100)
*D*_3_	(13 500, 16 000, 20 000)	(29 350, 58 700, 88 050)	(3 400, 6 800, 13 600)
*D*_4_	(1 800, 2 200, 2 500)	(6 300, 12 600, 18 900)	(400, 790, 1 580)
*D*_5_	(6 400, 8 400, 10 000)	(21 030, 42 060, 63 090)	(1 700, 3 400, 6 800)
*D*_6_	(1 800, 2 250, 2 500)	(15 100, 30 200, 45 300)	(400, 810, 1 620)

Due to the extremely serious situation of this rainstorm disaster, emergency materials reserved in Henan Province could not meet the demand of each disaster area. Therefore, temporary disaster relief reserves in Changsha, Shenyang, Xi’an, Lanzhou, Wuhan, Tianjin, Jiangsu, Beijing, and Zhengzhou, as well as organizations such as Henan Red Cross Society and the Henan Charity Federation are selected as rescue areas in this study, denoted by *S*_1_, *S*_2_, *S*_3_, *S*_4_, *S*_5_, *S*_6_, *S*_7_, *S*_8_, *S*_9_, *S*_10_, *S*_11_ respectively. The reserve quantity of emergency materials (bottled water, masks, and instant noodles) in these 11 rescue areas is shown in Table 2.

**Table 2:** The reserve quantity of emergency materials in each rescue area.

$x_{ij}^{k}$	*S*_1_	*S*_2_	*S*_3_	*S*_4_	*S*_5_	*S*_6_	*S*_7_	*S*_8_	*S*_9_	*S*_10_	*S*_11_
*R*_1_	4880	1815	5100	2000	3450	1248	3420	5000	29000	3650	100000
*R*_2_	1320	1350	4500	1000	1000	2000	790	4200	200000	1000	100000
*R*_3_	1250	1540	5400	2700	1200	1450	2560	3000	7200	4050	20000

Due to the suddenness and urgency of rainstorm disasters in Henan, it’s necessary to consider the uncertainty of dispatching cost and dispatching time when dispatching emergency materials. In this regard, triangular fuzzy number is used to represent the above variables. According to the above assumptions, the triangular fuzzy number of emergency materials dispatching cost from each rescue area to each disaster area is shown in Table 3, and the triangular fuzzy number of emergency materials dispatching time of from each rescue area to each disaster area is shown in Table 4.

**Table 3:** Triangular fuzzy number of dispatching cost from each rescue area to each disaster area.

$\tilde{c_{ij}}$	*D*_1_	*D*_2_	*D*_3_	*D*_4_	*D*_5_	*D*_6_
*S*_1_	(362, 393, 448)	(343, 379, 406)	(364, 390, 479)	(343, 379, 406)	(377, 406, 446)	(373, 394, 411)
*S*_2_	(561, 609, 620)	(581, 625, 640)	(587, 634, 646)	(581, 625, 640)	(595, 622, 633)	(623, 650, 662)
*S*_3_	(215, 226, 230)	(265, 269, 311)	(217, 226, 230)	(234, 242, 247)	(192, 205, 231)	(182, 200, 215)
*S*_4_	(450, 528, 530)	(681, 714, 717)	(452, 531, 541)	(753, 786, 860)	(420, 499, 512)	(421, 499, 513)
*S*_5_	(217, 228, 239)	(242, 262, 268)	(218, 236, 236)	(197, 225, 263)	(229, 250, 302)	(226, 234, 238)
*S*_6_	(290, 311, 315)	(310, 335, 340)	(317, 338, 342)	(310, 335, 340)	(321, 326, 329)	(330, 354, 357)
*S*_7_	(280, 282, 297)	(294, 306, 317)	(274, 285, 298)	(249, 272, 283)	(289, 303, 310)	(271, 284, 303)
*S*_8_	(299, 314, 320)	(458, 467, 489)	(325, 340, 347)	(319, 334, 340)	(311, 331, 334)	(339, 360, 363)
*S*_9_	(14.8, 26.8, 28.8)	(46, 46, 48)	(53.2, 53.2, 53.2)	(88.6, 88.6, 96.6)	(8, 8, 8)	(90.6, 90.6, 90.6)
*S*_10_	(12.8, 12.8, 12.8)	(82.4, 89.4, 89.4)	(24.8, 43.8, 43.8)	(20.4, 52.4, 52.4)	(22.8, 48.8, 55.8)	(81.6, 81.6, 81.6)
*S*_11_	(5.6, 5.6, 5.6)	(78.4, 78.4, 78.4)	(41, 41, 41)	(21.2, 21.2, 71.2)	(13.2, 39.2, 39.2)	(82.8, 82.8, 82.8)

**Table 4:** Triangular fuzzy number of dispatching time from each rescue area to each disaster area.

$\tilde{t_{ij}}$	*D*_1_	*D*_2_	*D*_3_	*D*_4_	*D*_5_	*D*_6_
*S*_1_	(767, 768, 536)	(500, 501, 513)	(512, 539, 583)	(500, 501, 513)	(491, 517, 524)	(464, 507, 518)
*S*_2_	(880, 1018, 1020)	(887, 1046, 1046)	(896, 1063, 1065)	(888, 1048, 1054)	(830, 825, 845)	(857, 862, 873)
*S*_3_	(314, 335, 340)	(180, 181, 187)	(311, 321, 327)	(337, 349, 353)	(273, 313, 324)	(266, 282, 317)
*S*_4_	(728, 744, 745)	(423, 493, 501)	(731, 759, 834)	(469, 548, 558)	(671, 689, 699)	(659, 676, 686)
*S*_5_	(334, 348, 364)	(345, 358, 366)	(328, 345, 351)	(298, 337, 338)	(326, 348, 362)	(312, 321, 324)
*S*_6_	(431, 437, 446)	(446, 452, 466)	(454, 463, 471)	(446, 453, 464)	(396, 419, 421)	(439, 446, 452)
*S*_7_	(412, 418, 420)	(431, 434, 442)	(410, 411, 413)	(382, 387, 388)	(398, 400, 401)	(387, 390, 405)
*S*_8_	(436, 443,446)	(326, 341, 344)	(455, 463, 466)	(448, 456, 459)	(417, 428, 433)	(444, 453, 459)
*S*_9_	(48, 50, 51)	(66, 67, 69)	(51, 66, 84)	(79, 82, 108)	(35, 44, 45)	(76, 80, 104)
*S*_10_	(40, 41, 42)	(78, 90, 101)	(69, 70, 72)	(65, 76, 79)	(68, 69, 75)	(91, 93, 100)
*S*_11_	(32, 35, 37)	(67, 70, 90)	(63, 73, 80)	(71, 77, 80)	(57, 65, 70)	(84, 87, 90)

In addition, the Amap App is used for distance measurement in this study, thus the distance from each rescue area to each disaster is shown in Table 5.

**Table 5:** The distance from each rescue area to each disaster area.

	*D*_1_	*D*_2_	*D*_3_	*D*_4_	*D*_5_	*D*_6_
*S*_1_	810	830	805	780	830	790
*S*_2_	1350	1400	1390	1380	1370	1420
*S*_3_	485	420	480	520	450	440
*S*_4_	1120	1050	1120	1150	1090	1075
*S*_5_	510	550	515	470	540	520
*S*_6_	710	765	750	724	720	790
*S*_7_	675	720	680	640	700	690
*S*_8_	680	740	725	710	705	760
*S*_9_	51	65	6	55	37	30
*S*_10_	22	87	60	54	55	95
*S*_11_	12	76	50	56	45	85

### 4.2 Data analysis

#### 4.2.1 Fuzzy determination of emergency material dispatching time and dispatching cost

When converting fuzzy number *μ*_M_(*x*) = (*l*, *m*, *μ*) into definite value, it’s necessary to comprehensively considerate the decision- maker’s preference and the eigenvalues of the triangular fuzzy number. Expected value, as another digital index to measure the size of fuzzy number value, provides an effective method for ordering fuzzy number, so as to avoid errors caused by decision-makers’ preference. For this reason, this study sets $\lambda =\frac{1}{2}$, and triangular fuzzy number of the overall expected value is $E(\mu_{M}(x))=\frac{1+2m+\mu }{4}$. For the convenience of subsequent model calculations, this study uses the defuzzification method of triangular fuzzy number to convert the emergency materials demand, dispatching time and dispatching cost into definite value, which are respectively shown in Tables 6–8.

**Table 6:** The overall expected value of emergency materials demand quantity in each disaster area.

$d_{i}^{k}$	*R*_1_	*R*_2_	*R*_3_
*D*_1_	112150	230020	47500
*D*_2_	1450	16000	620
*D*_3_	16375	58700	7650
*D*_4_	2175	12600	890
*D*_5_	8300	42060	3825
*D*_6_	2200	30200	910

**Table 7:** The overall expected value of dispatching cost from each rescue area to each disaster area.

*c*_*ij*_	*D*_1_	*D*_2_	*D*_3_	*D*_4_	*D*_5_	*D*_6_
*S*_1_	399	377	406	377	409	393
*S*_2_	600	618	625	618	618	646
*S*_3_	224	279	225	241	208	199
*S*_4_	509	707	514	796	483	483
*S*_5_	228	259	232	228	258	233
*S*_6_	307	330	334	330	326	349
*S*_7_	285	306	286	269	301	286
*S*_8_	312	470	338	332	327	356
*S*_9_	24.3	46.5	53.2	90.6	8	90.6
*S*_10_	12.8	87.65	39.05	44.4	44.05	81.6
*S*_11_	5.6	78.4	41	33.7	32.7	82.8

**Table 8:** The overall expected value of dispatching time from each rescue area to each disaster area.

*t*_*ij*_	*D*_1_	*D*_2_	*D*_3_	*D*_4_	*D*_5_	*D*_6_
*S*_1_	710	504	543	504	512	499
*S*_2_	984	1006	1022	1010	831	864
*S*_3_	331	182	320	347	306	287
*S*_4_	740	478	771	531	687	674
*S*_5_	349	357	342	328	346	320
*S*_6_	438	454	463	454	414	446
*S*_7_	417	435	411	386	400	393
*S*_8_	442	338	462	455	427	452
*S*_9_	49.75	67.25	66.75	87.75	42	85
*S*_10_	41	89.75	70.25	74	70.25	94.25
*S*_11_	34.75	74.25	72.25	76.25	64.25	87

The results of Table 6 show that, comparing to other disaster areas, *D*_1_ has the maximum demand for *R*_1_, *R*_2_ and *R*_3_. From the demand data of *R*_1_, *R*_2_ and *R*_3_, it can be seen that the emergency materials demand for each disaster area is directly proportional to the number of affected- population. That is to say, the more affected population at a certain disaster area, the relative increase in demand of emergency materials. Therefore, the emergency materials demand of these 6 disaster areas follows the order: *D*_1_>*D*_3_>*D*_5_>*D*_6_>*D*_4_>*D*_2_.

Table 7 shows the overall expected value of dispatching cost from each rescue area to each disaster area calculated based on the data in Table 3. From the results of Table 7, it can be seen that among the 11 rescue areas, *S*_1_−*S*_8_ are located outside Henan Province. The overall expected value of dispatching cost from these 8 rescue areas to each disaster area is greater than the cost value of dispatching emergency materials from the 3 rescue areas within the province *S*_9_, *S*_10_ and *S*_11_ to each disaster area (the data processing in Table 7 is mainly used for subsequent model calculation).

Similarly, Table 8 shows the overall expected value of dispatching time from each rescue area to each disaster area calculated based on the data in Table 4. From the results of Table 8, it can be seen that among the 11 rescue areas, *S*_1_−*S*_8_ are located outside Henan Province. The overall expected value of dispatching time from these 8 rescue areas to each disaster area also greater than the time of dispatching emergency materials from the 3 rescue areas within the province *S*_9_, *S*_10_ and *S*_11_ to each disaster area (the data processing in Table 8 is also used for subsequent model calculation).

#### 4.2.2 Determination of urgency coefficient for emergency material demand

According to the description of the urgency coefficient calculation for emergency material demand in sub-section 3.1, this study collected data of 5 evaluation indexes in 6 disaster areas, including the number of disaster-affected population, dead and missing population, emergency transfer and resettle population, damaged houses and direct economic loss. These data all obtained from the official website of China’s National Natural Disaster Management System, the investigation report released by the disaster investigation team of China’s State Council, and official reports issued by various cities in Henan Province, China [54]. Python is used to calculate the emergency material demand urgency coefficient *λ*_*i*_ of each disaster area in this study, as shown in Table 9.

**Table 9:** Evaluation indexes data and calculation results of urgency coefficient in each disaster area.

Disaster area (City)	Disaster-affected population (10,000 people)	Dead and missing population	Emergency transfer and resettle population (10,000 people)	Number of damaged houses	Direct economic loss (100 million)	Emergency materials demand urgency coefficient λ_i_
Zhengzhou	115.01	112	103.54	30106	145.51	21.60
Gongyi	6.3	84	2.3663	13000	70	2.02
Xinmi	29.35	58	5.36	56546	61.4	3.36
Xinzheng	8	17	4.5	8652	11.27	1.00
Xingyang	21.03	96	6.0541	25887	50.62	2.86
Dengfeng	15.1	13	1.2598	18793	27.2	1.28

#### 4.2.3 Model application

The emergency materials dispatching problem that considering the emergency material demand urgency belongs to a typical multi-objective optimization problem. Therefore, MATLAB is used to implementation the model which constructed in this study, and to calculate the dispatching quantity results of *R*_1_, *R*_2_ and *R*_3_, as respectively shown in Tables 10–12.

**Table 10:** Dispatching quantity results of *R*_1_.

$x_{ij}^{1}$	*D*_1_	*D*_2_	*D*_3_	*D*_4_	*D*_5_	*D*_6_
*S*_1_	4880	0	0	0	0	0
*S*_2_	1815	0	0	0	0	0
*S*_3_	5100	0	0	0	0	0
*S*_4_	2000	0	0	0	0	0
*S*_5_	3450	0	0	0	0	0
*S*_6_	1248	0	0	0	0	0
*S*_7_	3420	0	0	0	0	0
*S*_8_	5000	0	0	0	0	0
*S*_9_	29000	0	0	0	0	0
*S*_10_	3650	0	0	0	0	0
*S*_11_	52587	1450	16375	2175	8300	2200

**Table 11:** Dispatching quantity results of *R*_2_.

$x_{ij}^{2}$	*D*_1_	*D*_2_	*D*_3_	*D*_4_	*D*_5_	*D*_6_
*S*_1_	1320	0	0	0	0	0
*S*_2_	1350	0	0	0	0	0
*S*_3_	4500	0	0	0	0	0
*S*_4_	1000	0	0	0	0	0
*S*_5_	1000	0	0	0	0	0
*S*_6_	2000	0	0	0	0	0
*S*_7_	790	0	0	0	0	0
*S*_8_	4200	0	0	0	0	0
*S*_9_	92384	12800	32433	10080	25776	21034
*S*_10_	1000	0	0	0	0	0
*S*_11_	74477	0	14527	0	7871	3125

**Table 12:** Dispatching quantity results of *R*_3_.

$x_{ij}^{3}$	*D*_1_	*D*_2_	*D*_3_	*D*_4_	*D*_5_	*D*_6_
*S*_1_	1250	0	0	0	0	0
*S*_2_	1540	0	0	0	0	0
*S*_3_	5056	0	344	0	0	0
*S*_4_	2700	0	0	0	0	0
*S*_5_	1200	0	0	0	0	0
*S*_6_	1450	0	0	0	0	0
*S*_7_	2560	0	0	0	0	0
*S*_8_	3000	0	0	0	0	0
*S*_9_	5956	0	1244	0	0	0
*S*_10_	4050	0	0	0	0	0
*S*_11_	9243	495	4531	711	3059	727

The results of Table 10 show that, there is a high demand of *R*_1_ for *D*_1_. Except *S*_11_, the other 10 rescue areas dispatch *R*_1_ only to *D*_1_. *S*_9_ and *S*_11_ dispatch most of the demand of *R*_1_ to *D*_1_. In addition, due to the sufficient reserve of *R*_1_ in *S*_11_, *S*_11_ not only dispatches *R*_1_ to *D*_1_, but also dispatches an appropriate amount of *R*_1_ to the other 5 disaster areas.

The results of Table 11 show that, there is a high demand of *R*_2_ for *D*_1_. Except *S*_9_ and *S*_11_, the other 9 rescue areas dispatch *R*_2_ only to *D*_1_. *S*_9_ and *S*_11_ dispatch most of the demand of R_2_ for *D*_1_. In addition, due to the sufficient reserve of *R*_2_ in *S*_9_ and *S*_11_, these two rescue areas not only dispatch *R*_2_ to *D*_1_, but also dispatch an appropriate amount of *R*_2_ to other 5 disaster areas.

The results of Table 12 show that, the demand of *R*_3_ by *D*_1_ is the highest, the demand of *R*_3_ for *D*_3_ comes second. All 11 rescue areas dispatch an appropriate amount of *R*_3_ to *D*_1_, while *S*_3_, *S*_9_ and *S*_11_ dispatch an appropriate amount of *R*_3_ to *D*_3_. Due to the sufficient reserve of *R*_3_ in *S*_11_, the demand of *R*_3_ for the other 4 disaster areas *D*_2_, *D*_4_, *D*_5_ and *D*_6_ are all met by *S*_11_.

### 4.3 Results

From the above calculation results, it can be seen that when the objectives are the minimum dispatching time and dispatching cost, and the maximum dispatching fairness, the optimal emergency materials dispatching scheme for each disaster area is shown in Table 13.

**Table 13:** Emergency materials dispatching scheme for each disaster area.

*S*_*j*_	*D*_*i*_	R_1_	R_2_	R_3_
*S*_1_	*D*_1_	4880	1320	1250
*S*_2_		1815	1350	1540
*S*_3_		5100	4500	5056
*S*_4_		2000	1000	2700
*S*_5_		3450	1000	1200
*S*_6_		1248	2000	1450
*S*_7_		3420	790	2560
*S*_8_		5000	4200	3000
*S*_9_	*D*_1_	29000	92384	5956
	*D*_2_	0	12800	0
	*D*_3_	0	32433	1244
	*D*_4_	0	10080	0
	*D*_5_	0	25776	0
	*D*_6_	0	21034	0
*S*_10_	*D*_1_	3650	1000	4050
*S*_11_	*D*_1_	52587	74477	9243
	*D*_2_	1450	0	495
	*D*_3_	16375	14527	4531
	*D*_4_	2175	0	711
	*D*_5_	8300	7871	3059
	*D*_6_	2200	3125	727

The results in Table 13 shows that, the emergency materials required by *D*_2_, *D*_3_, *D*_4_, *D*_5_ and *D*_6_ are dispatched from *S*_9_ and *S*_11_. Due to the limited quantity of emergency materials available in 8 rescue areas outside Henan province, and the distance from these 8 rescue areas to disaster areas is much greater than the distance from 3 rescue areas inside Henan province to disaster areas. For this reason, all emergency materials in rescue areas *S*_1_−*S*_8_ and *S*_10_ are dispatched to D_1_. From the dispatching quantity results, it can be seen that the task of emergency materials dispatching for each disaster area will be completed by a certain rescue area, and dispatching quantity of *R*_1_ is greater than the total demand of 6 disaster areas. That is, it can meet 100% of emergency materials demand of all disaster areas. The remaining two types of emergency materials (*R*_2_ and *R*_3_) can meet 80% of the demand for 6 disaster areas. The results also show that, under uncertain situations, when comprehensively considering the objectives of dispatching time, dispatching cost, and dispatch fairness: 1) *S*_9_ and *S*_11_ can spend minimum dispatching time and dispatching cost to meet the emergency material demand of the other 5 disaster areas except *D*_1_; 2) *S*_9_ and *S*_11_ dispatch the remaining emergency materials reserves to *D*_1_; 3) *S*_1_−*S*_8_ and *S*_10_ undertake the task of dispatching the remaining emergency materials for *D*_1_. That is to say, the unmet demand of *D*_1_ is dispatched in time to achieve the optimal overall dispatching result and the highest satisfaction rate of emergency material demand for each disaster area.

In addition, the optimal emergency materials dispatching scheme shows that rescue areas *S*_3_, *S*_9_, and *S*_11_ involve emergency materials dispatching to multiple disaster areas. Therefore, this study uses ant colony algorithm to optimize transportation route based on MATLAB R2022a in the Win 11 operating system environment with 16GB (4800Mhz) of memory. Based on the calculation results of emergency materials dispatching, this study sets the parameters of the ant colony algorithm as shown in Table 14, so as to calculate the optimal scheme of emergency materials dispatching transportation route.

**Table 14:** Ant colony algorithm parameter setting.

number of ants	iterations	pheromone decay factor	Pheromone importance parameter	heuristic function importance parameter
20	100	0.5	1	2

**Fig 2:**
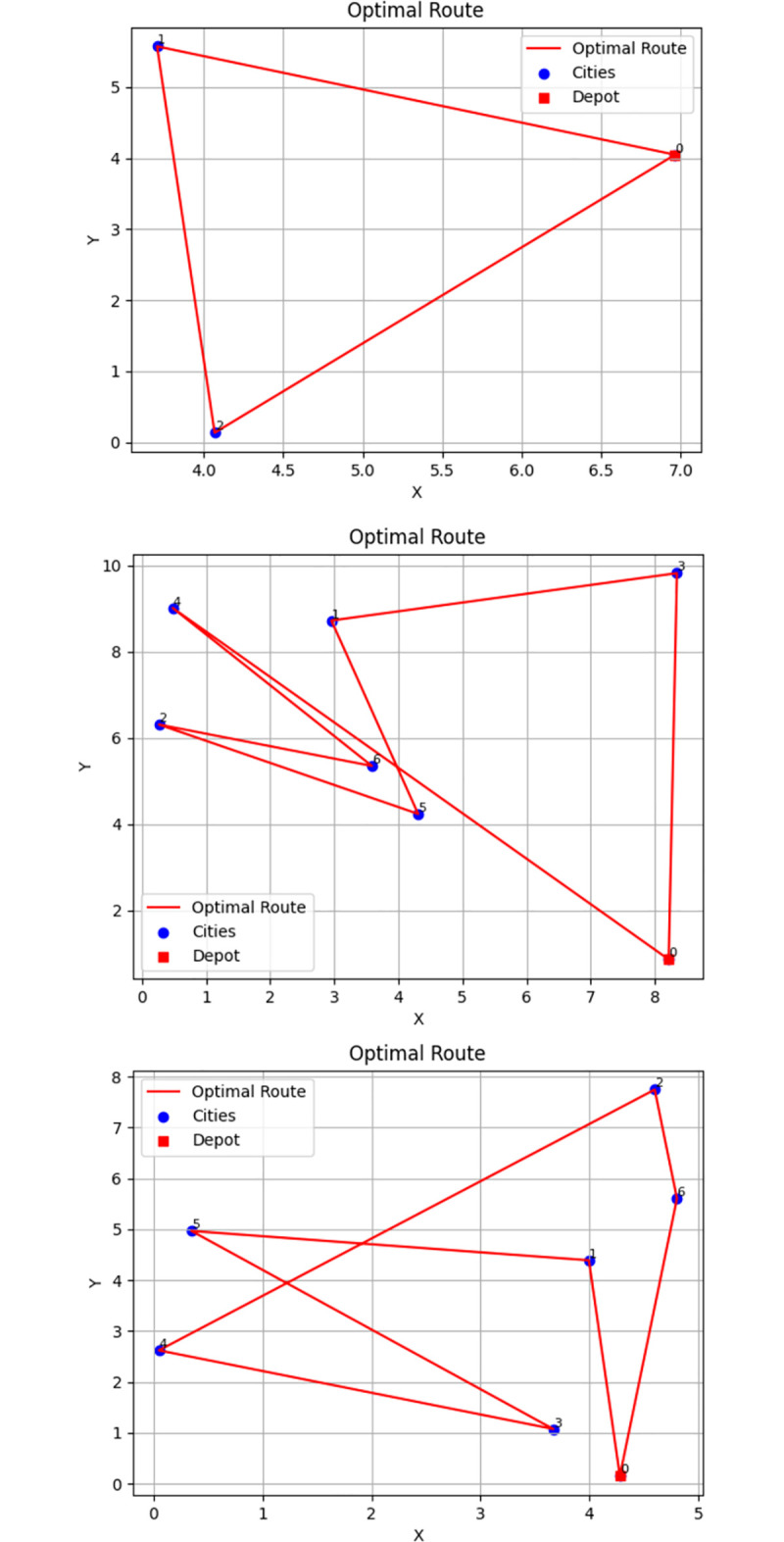
Optimal transportation route diagrams of *S*_3_, *S*_9_, and *S*_11_. (a) *S*_3_. (b) *S*_9_. (c) *S*_11_.

Through multiple iterations of the program calculation, the optimal scheme of emergency materials dispatching transportation route from the three rescue areas *S*_3_, *S*_9_ and *S*_11_ to each disaster area are finally output, as shown in Table 15. The optimal transportation route diagrams of *S*_3_, *S*_9_, and *S*_11_ are shown in Fig 2.

**Table 15:** Calculation results of the optimal transportation route and the shortest distance.

*S*_*j*_	Optimal transportation route	Shortest distance (km)
*S*_3_	*S*_3_→*D*_3_→*D*_1_	519.6
*S*_9_	*S*_9_→*D*_3_→*D*_1_→*D*_5_→*D*_2_→*D*_6_→*D*_4_	241.4
*S*_11_	*S*_11_→*D*_1_→*D*_5_→*D*_3_→*D*_4_→*D*_2_→*D*_6_	295.4

To sum up, ant colony algorithm with good operability is applied in this study, the optimal solution of emergency materials dispatching quantity is obtained by MATLAB. Then, the ant colony algorithm is used for route optimization to develop the optimal dispatching scheme, so as to achieve the objectives of the minimum dispatching time and dispatching cost, and the maximum dispatching fairness. This will help emergency management decision-makers to formulate effective decisions, so that providing support for similar major natural disasters in the future.

## 5. Discussion

**Fig 3:**
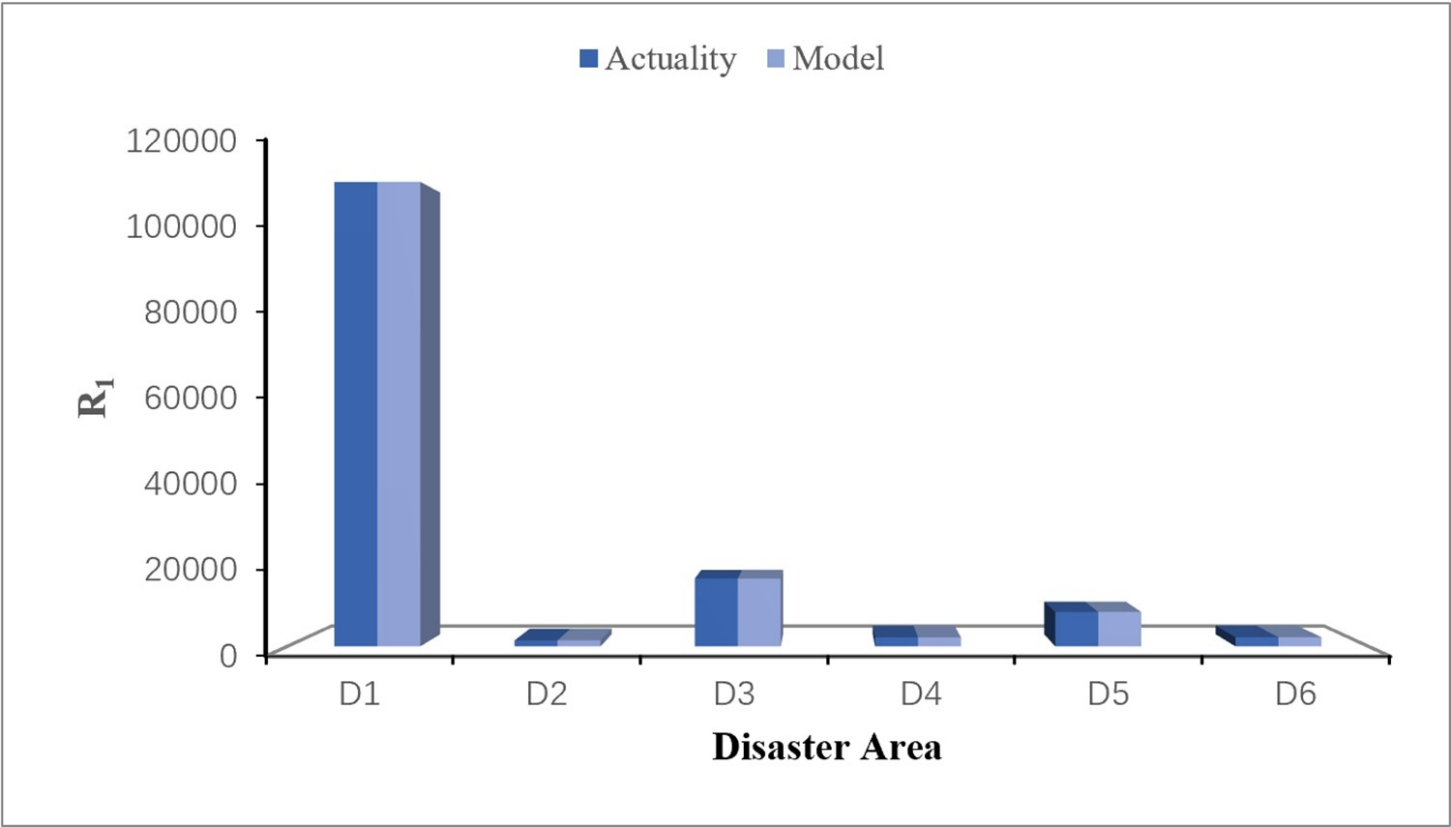
Comparison between actual demand of R_1_ and model results.

**Fig 4:**
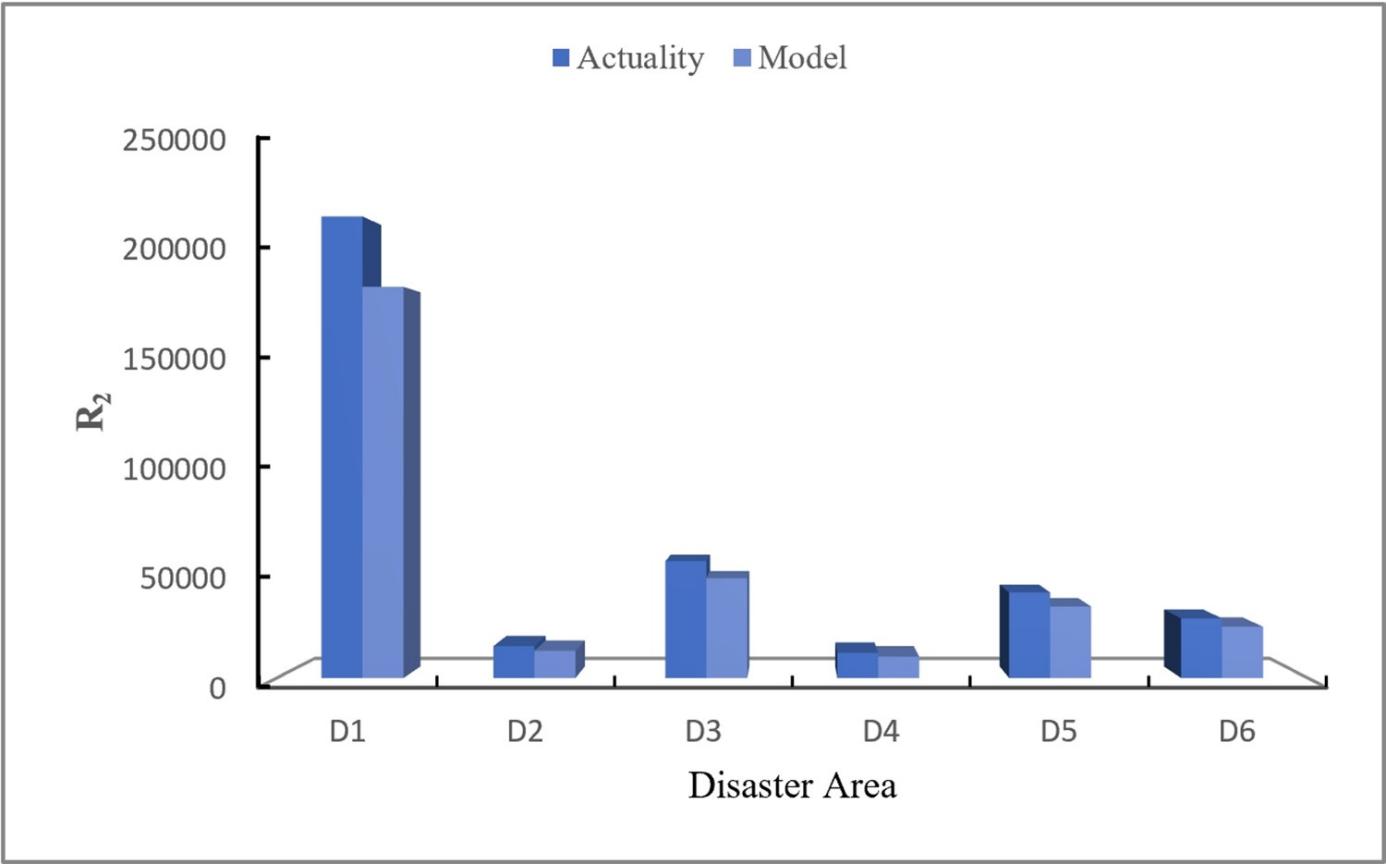
Comparison between actual demand of R_2_ and model results.

**Fig 5:**
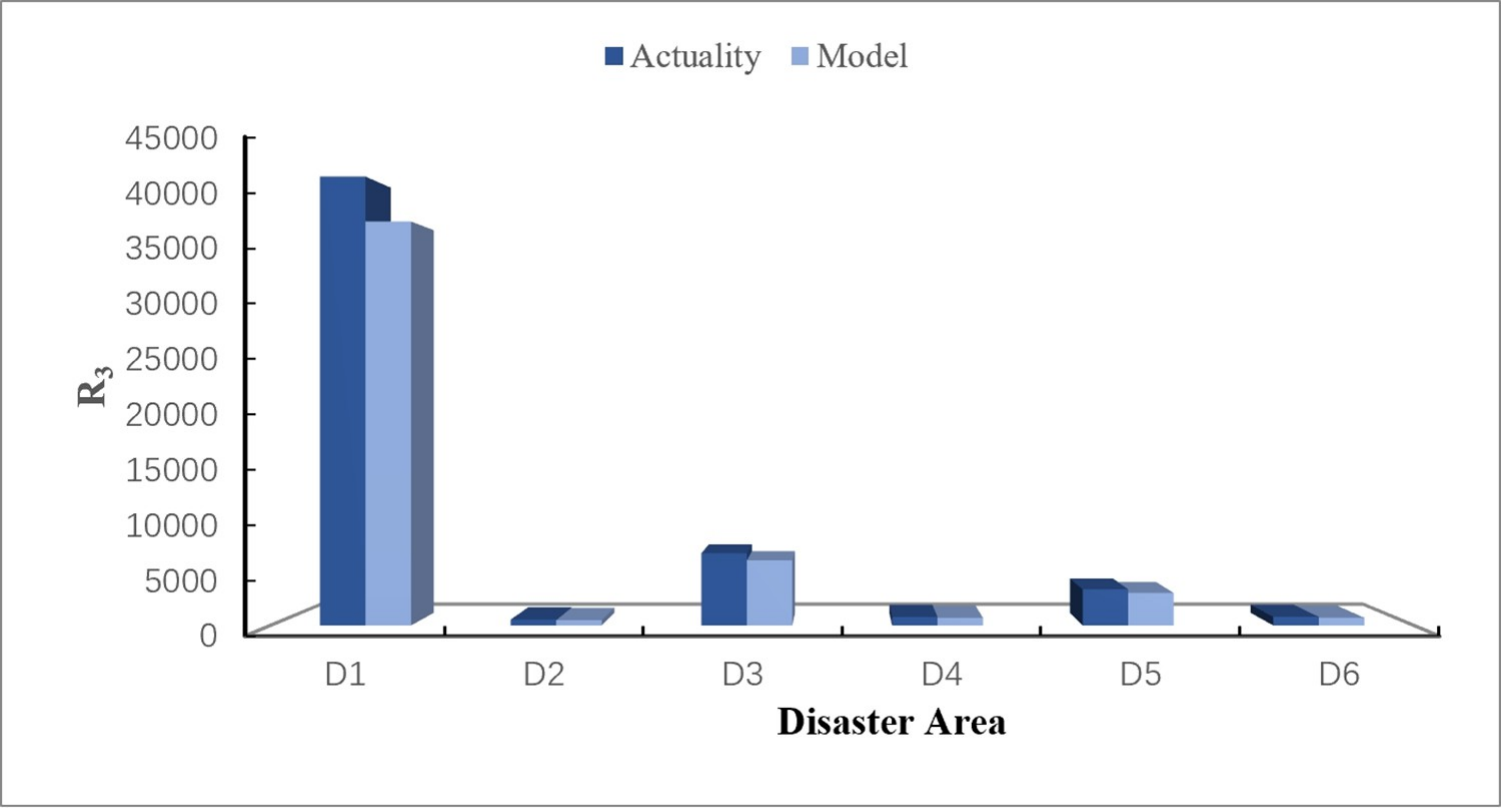
Comparison between actual demand of R_3_ and model results.

**Fig 6:**
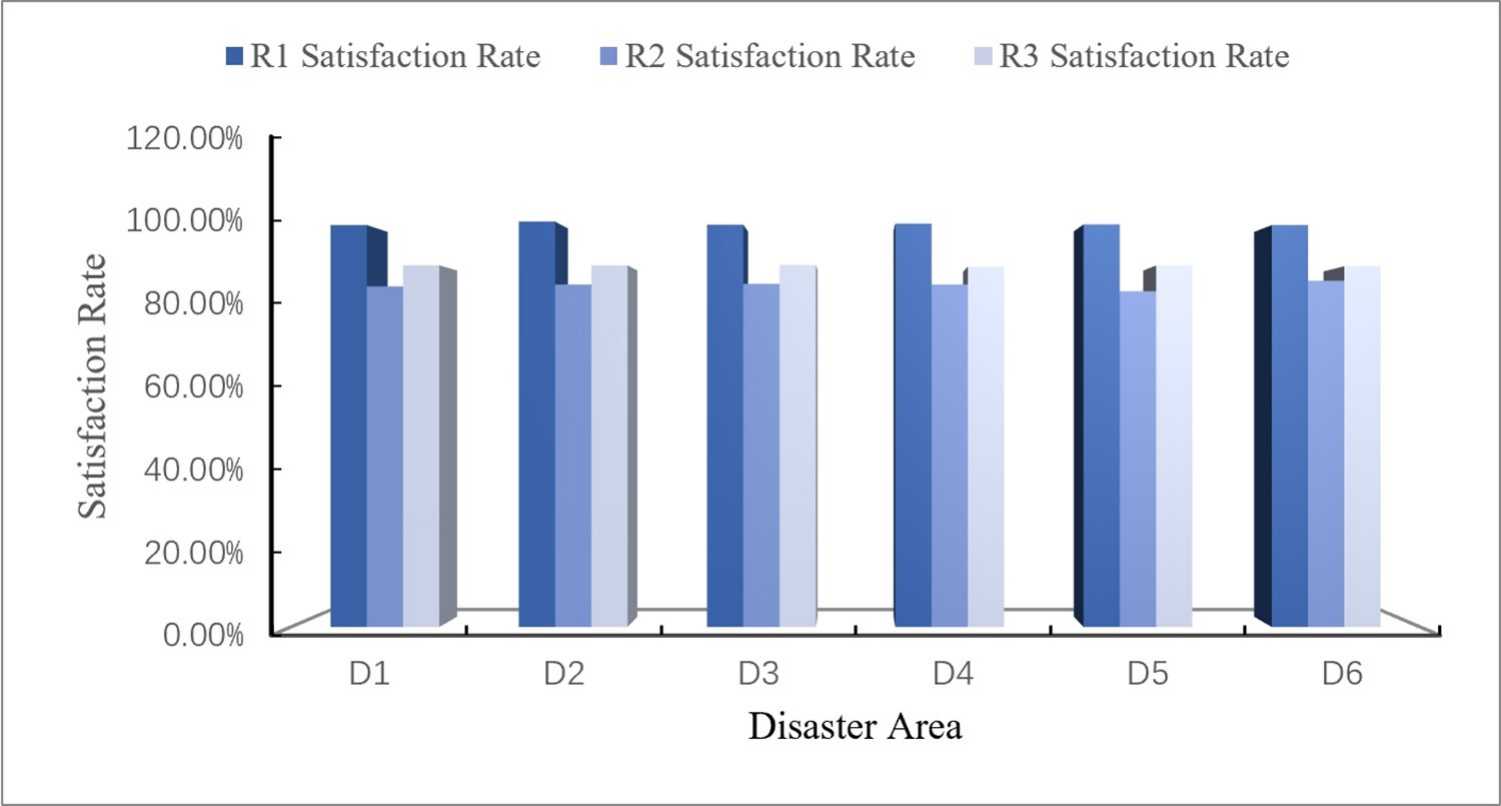
The emergency materials demand satisfaction rate of each disaster area.

The actual data of emergency materials demand required by each disaster area in this rainstorm disaster is obtained from the official websites of relevant departments (Red Cross Society of China), as shown in Table 16. Visualize comparison between the calculation results of the disaster case through CREMIDM-MND and the actual demand quantities of emergency materials required by each disaster area is conducted in this study, as shown in Figs 3–5. Through the comparison, it can be seen that the difference between the dispatching quantity results of *R*_1_ which calculated by CREMIDM-MND and the actual demand of *R*_1_ in each disaster area is very low, and it can basically achieve a 100% satisfaction rate for demand of *R*_1_. Although the actual demand of *R*_2_ and *R*_3_ is more than the emergency material dispatching calculation results by the model, in fact, the satisfaction rate of emergency materials demand for disaster areas has reached over 80%, as shown in Fig 6. Meanwhile, the results also show that the emergency materials demand satisfaction rate of each disaster area is balanced, which can ensure the maximum dispatching fairness of each disaster area while basically meeting the demand.

**Table 16:** The actual demand of emergency materials in each disaster area.

disaster area	R_1_	*R*_2_	*R*_3_
*D*_1_	112 132	217 097	42 233
*D*_2_	1 437	15 021	550
*D*_3_	16 359	55 000	6 793
*D*_4_	2 167	11 830	793
*D*_5_	8 286	40 280	3 400
*D*_6_	2 200	28 047	810

Fig 3 shows that, for the emergency material *R*_1_, by comparing the actual emergency materials demand of disaster areas with the emergency materials dispatching scheme solved by the model constructed in this study, it can be seen that the actual emergency materials demand of disaster areas is basically the same as the dispatching results calculated by CREMIDM-MND, which can satisfy 100% of demand for disaster areas. In addition, the reserve of *R*_1_ in each rescue area is sufficient, so that can effectively meet the demand of *R*_1_ for each disaster area in a short period of time.

Fig 4 shows that, for the emergency material *R*_2_, by comparing the actual emergency materials demand of disaster areas with the emergency materials dispatching scheme solved by CREMIDM-MND, it can be seen that although the actual emergency materials demand of disaster areas is larger than the dispatching results calculated by CREMIDM-MND, which can satisfy 80% of the demand for disaster areas. It confirms that the results meet the model assumptions and can roughly satisfy the emergency material demand of all affected areas.

Fig 5 shows that, for the emergency material *R*_3_, similar to the case of *R*_2_, although the actual emergency materials demand of disaster areas is larger than the dispatching results calculated by CREMIDM-MND, which can still satisfy 80% of the demand for disaster areas. It confirms that the results also meet the model assumptions and can roughly satisfy the emergency material demand of all disaster areas.

Fig 6 shows a visual representation of the demand satisfaction rate for *R*_1_, *R*_2_ and *R*_3_ in each disaster area calculated by CREMIDM-MND. From Fig 6, it can be seen that the demand satisfaction rate of emergency materials in each disaster area reaches more than 80% and basically maintain balanced. Therefore, this result confirms that the dispatching scheme calculated through CREMIDM-MND can ensure the maximum dispatching fairness for all disaster areas while basically satisfying the emergency materials demand of all disaster areas.

**Fig 7:**
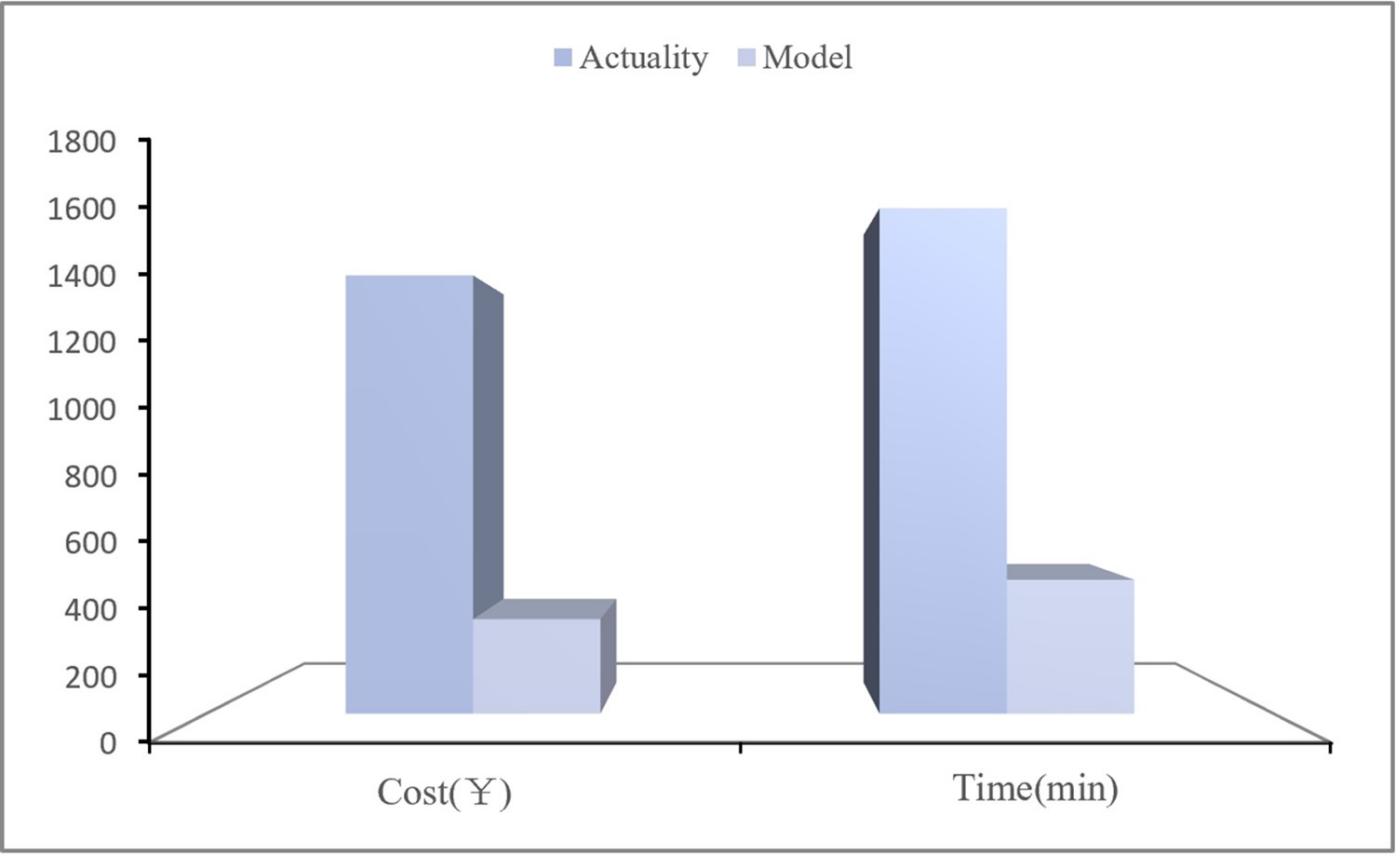
Comparison of *S*_8_’s actual dispatching cost and dispatching time with model calculation results.

In addition, taking the rescue area *S*_8_ as an example, the actual data of emergency materials dispatching time and dispatching cost are compared with the calculation results obtained by the CREMIDM-MND applying to the case, as shown in Fig 7. The results confirm that the dispatching cost and dispatching time obtained by CREMIDM-MND are much lower than the actual dispatching cost and dispatching time in the dispatching process.

The above results show that, compared with the actual dispatching data, the dispatching results calculated based on CREMIDM-MND can better meet the objectives of the minimum dispatching time and dispatching cost, and the maximum dispatching fairness for disaster area. The results also confirm that the model proposed in this study has high reliability, and the dispatching scheme based on this model has certain reference value for dealing with similar disasters in the future.

## 6. Conclusions

Emergency materials dispatching is a very important job in emergency rescue in major natural disasters. As a complex multi-objective optimization problem, it brings challenges and difficulties to the government emergency rescue activities. In this study, we make efforts to construct CREMIDM-MND for solving the problem. The main work of this paper is summarized as follows. Firstly, considering the disaster severity level varies among different disaster areas, the urgency of emergency material demand is also different. The entropy weight method is used to determine the urgency coefficient of emergency material demand for each disaster areas in this study. Secondly, comprehensively taking into account the challenges faced by cross-regional emergency materials dispatching and the highly uncertain characteristics of emergency rescue in major natural disasters, this study aims to minimize the dispatching time and dispatching cost, and maximize the dispatching fairness for all disaster areas. The triangular fuzzy number method is used to represent the above variables, so that CREMIDM-MND is constructed. Then, the extremely heavy rainstorm disaster in Henan Province of China in 2021 is selected as a typical case, and CREMIDM-MND is applied to the case. The objective disaster relief data is obtained through the official website, and MATLAB is used to calculate the model. Ant colony algorithm is further used to optimize the transportation route of emergency materials dispatching, so that the cross-regional emergency material dispatching scheme is developed. Finally, this study visualized and compared the research results with the actual quantity of emergency materials required in each disaster area. The results confirm that the emergency material dispatching scheme based on CREMIDM-MND can better meet the emergency materials demand of disaster areas within a shorter time and a lower cost as well as ensure the fairness, so as to achieve reasonable dispatching of emergency materials across regions.

This study constructs CREMIDM-MND, which has significant management implications for the government to improve emergency rescue performance, so that to maximize the safety of people’s lives and reduce the economic losses caused by major natural disasters. On the one hand, for relevant government departments, when major natural disasters occur suddenly, they should quickly organize emergency rescue activities, obtain sufficient disaster data, and ensure timely and on-demand dispatching of emergency materials to the disaster areas in the shortest possible time. Therefore, the model constructed in this study can meet the emergency materials demand of disaster areas in the shortest time while minimizing dispatching costs, thus providing decision-making support for the government to formulate effective emergency material dispatching scheme. On the other hand, in order to successfully respond to large-scale disasters, it’s necessary to make the reasonable reserve of emergency materials within the region, built emergency logistics systems between adjacent regions, establish a scientific cross-regional emergency materials allocation system, and coordinate with surrounding areas to cope with disasters, which is the future development trend of emergency management. Therefore, the model constructed in this study provides emergency management support for cross-regional collaborative rescue to major natural disasters.

Although CREMIDM-MND balances the issue of time, cost, and fairness, emergency material dispatching in major natural disasters is a very complex problem in reality. Therefore, in addition to the above three aspects, other relevant factors should also be included in the research on this problem. In future work, we will conduct more in-depth research based on the existing foundation. For example, to address the dynamic demand for emergency materials, a multi-stage emergency material dispatching model will be studied. When faced with limited transportation capacity, the balance between demand and capacity will be taken into consideration in the research. In addition, the research object will be expanded, other types of natural disasters will be selected, in order to further improve the universality and stability of the constructed model.
